# Mobile Apps for Mental Health Issues: Meta-Review of Meta-Analyses

**DOI:** 10.2196/17458

**Published:** 2020-05-29

**Authors:** Tania Lecomte, Stéphane Potvin, Marc Corbière, Stéphane Guay, Crystal Samson, Briana Cloutier, Audrey Francoeur, Antoine Pennou, Yasser Khazaal

**Affiliations:** 1 Department of Psychology University of Montreal Montreal, QC Canada; 2 Centre de recherche l'Institut Universitaire en Santé Mentale de Montréal Montreal, QC Canada; 3 Department of Psychiatry University of Montreal Montreal, QC Canada; 4 Department of Education Career Counselling University du Quebec a Montreal Montreal, QC Canada; 5 Department of Criminology University of Montreal Montreal, QC Canada; 6 Department of Psychiatry Centre Hospitalier Universitaire Vaudois Lausanne Switzerland

**Keywords:** apps, mental health, depression, anxiety, review, meta

## Abstract

**Background:**

Mental health apps have great potential to help people needing support to cope with distress or specific symptoms. In fact, there is an exponential increase in the number of mental health apps available on the internet, with less than 5% being actually studied.

**Objective:**

This study aimed to assess the quality of the available evidence regarding the use of mental health apps and to summarize the results obtained so far.

**Methods:**

Systematic reviews and meta-analyses were searched, specifically for mobile apps on mental health issues or symptoms, and rated using the Grading of Recommendations Assessment, Development and Evaluation system.

**Results:**

A total of 7 meta-analyses were carefully reviewed and rated. Although some meta-analyses looked at any mental health issue and analyzed the data together, these studies were of poorer quality and did not offer strong empirical support for the apps. Studies focusing specifically on anxiety symptoms or depressive symptoms were of moderate to high quality and generally had small to medium effect sizes. Similarly, the effects of apps on stress and quality of life tended to offer small to medium effects and were of moderate to high quality. Studies looking at stand-alone apps had smaller effect sizes but better empirical quality than studies looking at apps with guidance. The studies that included follow-ups mostly found a sustained impact of the app at an 11-week follow-up.

**Conclusions:**

This meta-review revealed that apps for anxiety and depression hold great promise with clear clinical advantages, either as stand-alone self-management or as adjunctive treatments. More meta-analyses and more quality studies are needed to recommend apps for other mental health issues or for specific populations.

## Introduction

### Mobile Health and Apps

Recent years have seen an exponential development of mobile technologies aimed at improving various mental health problems. Such technologies are considered part of a new field of medicine called mobile health (*mHealth*). This term refers to health (including mental health) supported by mobile technologies [1]. The mHealth field is booming, with a plethora of health-related apps, websites, and text messaging–support interventions being developed by the industry and being adopted by the public [2]. However, only a small proportion of these technologies have undergone any form of empirical assessment [3].

This lack of app validation is a concern, even more so when studies suggest that mental health– and addiction-related apps currently available to the public, with few exceptions, offer insufficient content quality [4-8]. Fortunately, recent years have seen an increase in the gathering of empirical data related to smartphone app–related interventions [9].

### What Are Mobile Mental Health Interventions?

According to the World Health Organization’s definition of mHealth, mobile mental health interventions could be considered as mental health services (medical and public health practices) “supported by mobile devices, such as mobile phones, patient monitoring devices, personal digital assistants (PDAs), and other wireless devices” [10]. They include smartphone apps, voice, video or text messaging interventions, real-time tracking, and Web-based interventions, to name a few. In this review, we were specifically interested in mental health apps.

Smartphone apps, because of their worldwide mobility, connectivity, 24-hour availability, and their ubiquitous characteristics, are strong vectors for mHealth interventions. Furthermore, they can convey a large range of technologies and functionalities, such as virtual reality, augmented reality (inserting computer elements into the real field), telemedicine, robotics, games, interfaces connected to sensors, social networks, real-time interactivity, geolocation, and more [11].

App technology has shown the greatest reach in the past few years. According to the United Nations, more than 90% of the population in developed countries use such apps daily [12]. Once available for the target audience, because of the wide dissemination of smartphone devices, such tools can attract many downloads from all over the world. This potential is illustrated by tens of thousands of downloads of such apps [13,14].

The ubiquitous, handy mobile format and 24-hour availability of smartphones offer an important advantage for using apps to target mental health problems. One may hypothesize that the treatment of mental disorders could be improved by effective support in the right place at the right time. As learning is context dependent [15], apps can support the process of empowerment and recovery of people with various mental health problems by allowing people to access tools or support when needed [16,17].

Mental health apps can also offer the opportunity to assess, with Ecological Momentary Assessment, or intervene, via Ecological Momentary Interventions (EMIs), individuals in their natural environment, thereby enabling a better understanding of the factors triggering problems and addressing the problems when and where they arise [18-20]. These methods overcome memory biases by asking questions pertaining to the current moment or the current day and can also help determine if phenomena are stable or change from day to day [21]. Furthermore, such ecologically valid data may help to guide treatments or improve assessments in naturalistic settings [22].

### Current Knowledge on Mobile Apps for Mental Health Problems

There is currently somewhere between 10,000 and 20,000 *mental health* apps [23,24], but it is estimated that only about 3% to 4% are actually evidence based. Most of these studies have been conducted in the last few years and assessed either the feasibility and acceptability of mental health apps and, in some cases, their efficacy for a broad spectrum of mental disorders, including depressive disorders, posttraumatic stress disorders, schizophrenia, bipolar disorders, or addictions [9,14,25-38].

Given the heterogeneity and speed of publication of app-related studies, aggregated results are needed to determine the overall (vs specific) efficacy of mobile apps for mental health. Multiple meta-analyses on apps focusing on a single or multiple mental health problems have been conducted [39,40], with very different results at times. This could be explained by the selection criteria for the meta-analyses, with some only focusing on stand-alone apps, others only looking at adjunctive apps (apps offered on top of another treatment) or apps offered with guidance (a person available for questions or to prompt its use), others considering both models together, and others still including everything and evaluating the models separately in different subanalyses. In fact, some authors suggest that only adjunctive apps or apps with guidance should be recommended at this point for mental health issues [41]. Given the speed of uptake of many of these apps, it is important to determine, based on the quality of the evidence available and the effect sizes, if we should recommend such apps for mental health problems such as depression or anxiety. The purpose of this meta-review was to summarize these results and determine the empirical quality of the evidence reported using the grading of recommendations, assessment, development, and evaluation (GRADE) system [42]. This system permits the quality of evidence produced by meta-analyses to be evaluated, according to specific factors: the sample size, the stable findings across studies, the appropriate control for known confounding factors, no evidence of study bias, follow-up (if any), and results being closely linked to the outcomes targeted here (see Tables 1 and 2). The GRADE system has been successfully applied to meta-analyses of pre-post designs, randomized controlled trials (RCTs), correlational studies, experimental studies, and longitudinal studies [42].

**Table 1:** GRADE review of included meta-analyses.

Authors	Technology used	Intervention type and subtype or outcome type	Effect size	Precise (less than 0.25 = precise)	Consistent	Direct
			d, g, OR, RR	95% CI, Yes or No	Q or I², Yes or No	Yes or No
**Lindhiem et al., 2015**	mHealth^a^ for psychotherapy or behavioural interventions (across all problems and issues)	Specifically for Apps (excluding PDAs), all mental health problems together	d= 0.57	95% CI: 0.28-0.85 - No	Q= 125.15, *P*>.001, (overall, not specific for apps)	No (multiple outcomes combined)
						
**Versluis et al., 2016**	Ecological momentary interventions (EMI) for anxiety, depression, stress and positive mental health	Ecological Momentary Interventions (EMIs); outcome type : global mental health, outliers removed	g=0.57	95% CI: 0.45-0.70, Yes	I²: 65.08, No	No (multiple outcomes)
	N/A^b^	EMIs; outcome type : global mental health compared to control conditions	g=0.65	95% CI : 0.48-0.82, No	I²: 58.5, No	No
	N/A	EMIs; outcome: anxiety	g=0.47	95% CI: 0.32-0.63 No	I²=50.48; No	Yes
	N/A	EMIs; outcome : depression	g=0.48	95% CI: 0.34-0.61 No	I²=65.58; No	Yes
	N/A	EMIs; outcome : perceived stress	g=0.40	95% CI : 0.23-0.57 No	I²= 12.79; Yes	No
	N/A	EMIs; outcome : quality of life	g= 0.38	95% CI : 0.19-0.56 No	I²=0; Yes	No
	N/A	with guidance	g=0.73	95% CI : 0.57-0.88 Yes	I²=37.1% Q=20.67; Yes	Yes
	N/A	stand alone	g= 0.45	95% CI : 0.22-0.69 No	I²=77.7% Q=36.8, *P*=.05; No	Yes
						
**Stratton et al., 2017**	eHealth and mHealth (app or web-based) for mental health at work	eHealth in the work place; outcome : global (No specific analyses just for apps)	g=0.24	95% CI: 0.13-0.35 (No)	I²: 67.6%, Q = 9.82 (df2), *P*<=.01, No	No (eHealth^c^ Smartphone; multiple outcomes)
						
**Firth et al., 2017a**	Psychological interventions for anxiety via smartphones	Smartphone interventions, all studies, anxiety symptoms	g=0.33	95% CI: 0.17-0.48 (No)	Q=15.9, I²=49.6%), No	Yes (anx)
	N/A	compared to active controls	g=0.19	0.06-0.31, Yes	Yes	Yes
	N/A	compared to waitlist	g=0.45	0.3-0.6, No	Yes	Yes
**Firth et al., 2017b**	Psychological interventions for depression via smartphones	Smartphone interventions for depression, global	g = 0.38	95% CI: 0.242 - 0.524, Yes	Q = 80.8 *P*=.01 I²=74%, No	Yes
	N/A	compared to active controls	g = 0.22	95% CI: 0.098 - 0.334, Yes	Q = 20.8 *P*=.03 I²=47.2, No	Yes
		compared to inactive controls	g = 0.56	95% CI: 0.379 - 0.736, No	Q =34.9 *P*=.01 I²=65.6, No	Yes
**Witt et al., 2017**	eHealth and mHealth (app or web-based) for self-management of suicidal ideation and self-harm – (stand alone only)	suicidal ideation RCT^d^	d=-0.26	95% CI: -0.44- -0.08, No	I²: 0%, Yes	Yes (suicidal ideation)
	N/A	suicidation ideation (No controls)	d=-0.4	95% CI = -0.92, 0.12 Yes	*P*=.003, I²: 79%, No	Yes
	N/A	self-harm (frequency) vs control	mean difference: 0.34	95% CI: -2.1 - 2.78, No	I² = 12%, *P*=.39, Yes	Yes
						
**Linardon et al. 2019**	apps	Apps for depression	g= 0.28	95% CI: 0.21-0.36 Yes	I² = 54%, No	Yes (depr sx)
	N/A	compared to active controls	g=0.13	95% CI: 0.07-0.34, No	I² = 60%, No	Yes
	N/A	with guidance	g=0.48	95% CI: 0.34-0.62 No	I² = 46%, No	Yes
	N/A	stand alone	g=0.23	95% CI: 0.15-0.31 No	I² = 32%, Yes	Yes
	N/A	Apps for anxiety	g=0.3	95% CI: 0.2-0.4, No	I² = 63%, No	Yes
	N/A	compared to active controls	g=0.09	95% CI: -0.21-0.39, Yes	I² = 32%, Yes	Yes
	N/A	with guidance	g=0.53	95% CI: -0.36-0.70, No	I² = 60%, No	Yes
	N/A	stand alone	g=0.21	95% CI: -0.12-0.30, No	I² = 36%, Yes	Yes
	N/A	Apps for social anxiety	g=0.58	95% CI: 0.25-0.90) No	I²: 78%, No	Yes
	N/A	Apps for panic	g=-0.05	95% CI: -0.41- 0.31, No	I²: 0% Yes	Yes
	N/A	Apps for PTSD	g=0.18	95% CI: -0.04-0.41 - No	I²: 0% Yes	Yes
	N/A	outcome: general distress	g=0.40	95% CI: 0.24-0.56, Yes	I²: 60% No	No
	N/A	outcome: stress	g=0.35	95% CI: 0.21-0.48, No	I² = 62%, No	No
	N/A	outcome: quality of life	g=0.35	95% CI: 0.29-0.42, Yes	I² = 24%,Yes	No

^a^mHealth: mobile health.

^b^N/A: Not applicable.

^c^RCT: Randomized Controlled Trial.

^d^eHealth: electronic health.

**Table 2:** Grade review continued.

Authors	Study Design	N (total number of participants)	Length of follow-up	Other biases considered	Publication bias considered	Conclusion on effect	Overall Quality
	RCT^a^, PC (prospective cohort)	P. eg.: 154, controls 145	No follow-up described	No, but they looked if the effect vary by moderator (supported by a mental health professional)	P. eg. : Yes, small - Yes, No change - No publication bias - unknown		
**Lindhiem et al., 2015**	RCT	943	No follow-up described	No	Yes: No change but not specific for apps	medium	poor-moderate
							
**Versluis et al., 2016**	PC	1008	No follow-up described	Yes (age, gender, design, etc.)	Yes, No change	medium	poor-moderate
	RCT	481	No follow-up described	No	Yes, significant risk for bias. Corrected effect size: g=0.23, 95% CI: 0.04-0.42 (considerably smaller)	medium	poor
	PC +RCT	468	No follow-up described	No	unknown for this subgroup of studies	small-medium	poor
	PC	870	No follow-up described	No	unknown for this subgroup of studies	small-medium	poor
	PC	199	No follow-up described	No	unknown for this subgroup of studies	small-medium	poor
	PC	1156	No follow-up described	No	unknown for this subgroup of studies	small-medium	poor-moderate
	PC+RCT	474	No follow-up described	No	Unknown for this subanalysis	medium-large	moderate
	PC+RCT	425	No follow-up described	No	Unknown for this subanalysis	medium	poor
							
**Stratton et al., 2017**	RCT	2399, controls 2265 (total but only 3 app studies)	follow-up described (g=0.23), but not specific for apps	No	Yes, adjusted effect size = g=0.12, 95% CI = 0.01-0.25	small	poor-moderate
							
**Firth et al., 2017a**	RCT	960 (intervention conditions), 877 (control conditions)	No follow-ups described	No	No bias	small-medium	moderate-high
	RCT	total: 1026	No follow-ups described	No	No bias	small	high
	RCT	total:1212	No follow-ups described	No	No bias	small-medium	moderate-high
**Firth et al., 2017b**	RCT	1716, controls 1698	No follow-up described	Yes (CBT^b^/Not, mindfulness, feedback, person feedback, etc)	No bias *P*=.26	small-medium	high
	RCT	1195, controls 1186	No follow-up described	Yes	No bias *P*=.34	small	moderate-high
	RCT	891, controls 783	No follow-up described	Yes	No bias *P*=.25	medium	moderate-high
**Witt et al., 2017**	RCT	232, controls 236	2 studies: -0.34 (CI: -0.70-0.01)	No	unknown	small	moderate
	PC	149	No follow-up described	No	unknown	small-medium	poor
	RCT	104, controls: 121	No follow-up described	No	unknown	N.S.^c^	poor-moderate
							
**Linardon et al. 2019**	RCT	3639, controls: 3519	2-6 weeks (n=33): g=0.17, 7-11 weeks (n=18): g=0.46, 12+ (n=3): g=0.09	Yes, multiple subanalyses	Yes, results improved when controlled (g=0.41)	small-medium	high
	RCT	526, controls: 530		Yes, multiple subanalyses		small	moderate-high
	RCT	978, controls: 918				medium	poor-moderate
	RCT	2489, controls: 2522				small	moderate
	RCT	2219, controls: 2256	2-6 weeks (n=24): g=0.11, 7-11 weeks (n=15): g=0.52, 12+ (n=0)	Yes, multiple subanalyses	Yes, No change	small-medium	moderate-high
	RCT	134, controls: 137	No	Yes, multiple subanalyses	Yes, no change	N.S.	moderate-high
	RCT	859, controls: 827				medium	poor-moderate
	RCT	859, controls: 860				small	moderate
	RCT	520, controls: 326	No	Yes, low risk of bias	unknown	medium	poor-moderate
	RCT	58, controls: 56	No	Yes, low risk of bias	unknown	N.S.	poor-moderate
	RCT	145, controls: 147	No	Yes, low risk of bias	unknown	N.S.	poor-moderate
	RCT	919, controls: 949	No	Yes, low risk of bias	unknown	small-medium	moderate
	RCT	1574, controls: 1711	2-6 weeks (n=19): g=0.18, 7-11 weeks (n=6): g=0.63, 12+ (n=2): g=0.59	Yes, multiple subanalyses	Yes, results improved when controlled (g=0.44)	small-medium	moderate-high
	RCT	2714, controls: 2871	2-6 weeks (n=31): g=0.35, 7-11 weeks (n=11): g=0.36, 12+ (n=1): g=0.31	Yes, multiple subanalyses	Yes, results improved when controlled (g=0.39)	small-medium	high

^a^RCT: Randomized Controlled Trial.

^b^CBT: Cognitive Behavioral Therapy.

^c^N.S.:Not significant.

## Methods

### Literature Search

We only included systematic reviews reporting quantitative pooled data (ie, meta-analyses), published in full text, in English or French, and those mentioned the use of app technology for mental health issues.

When more than one meta-analysis was found for a mental health problem, we reviewed them all and used the following criteria to select the ones we kept: (1) if most of the same studies were reviewed, we kept the meta-analysis with the largest number of studies; and (2) between an older meta-analysis with many small uncontrolled studies and a more recent meta-analysis including only RCTs, we chose the latter. We excluded systematic reviews without quantifiable data (eg, qualitative) and treatment guidelines. The final decision to include or exclude reviews was made by consensus by 2 researchers (TL and SP).

### Search Strategy

**Figure 1:**
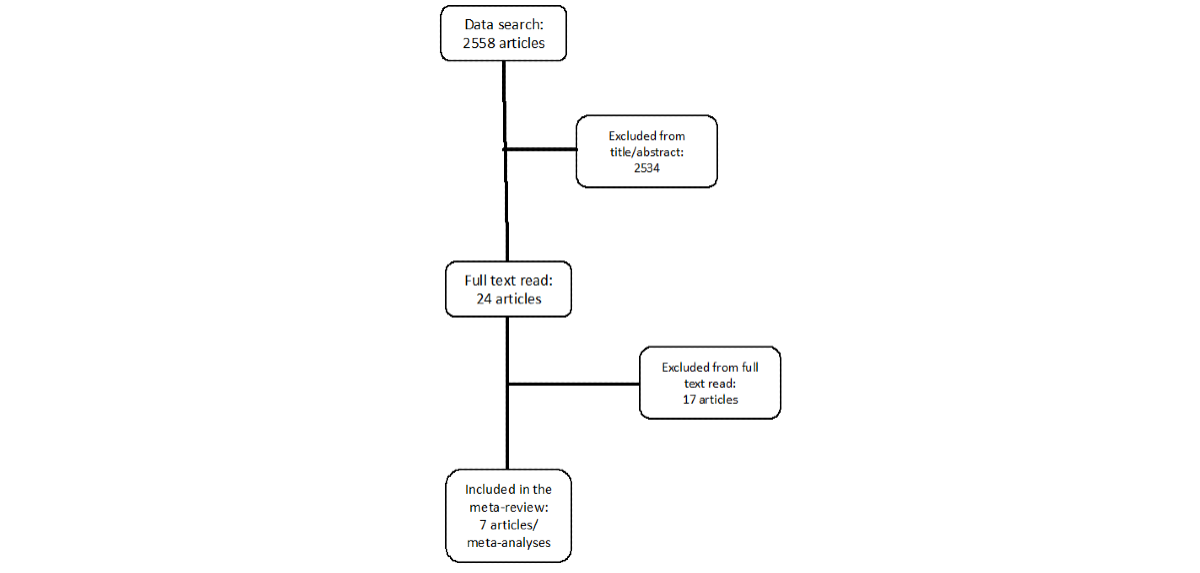
Flow diagram.

MEDLINE, EMBASE, Current Contents, PsycINFO, and Google Scholar were searched. Keywords included *mental health*, *technology*, *app*, *mHealth*, *eHealth*, *mobile*, with the added filters: *review* or *meta*. See Figure 1 for the selection of studies.

### Grading of Recommendations, Assessment, Development, and Evaluation System

The GRADE system was used to assess evidence quality [42]. According to this assessment system, the quality of evidence of meta-analyses can be judged based on various factors, namely, the size of the sample (the larger the better, ideally over 1000), the precision of effects (ie, the CI is not too wide; we opted for within 25% higher or lower than the effect size as ideal), the directness of the outcomes (eg, impact on mental health symptoms [direct] vs impact on perceived stress [indirect]), homogeneity of effects across studies (ie, consistency of results from one study to the next), the study design (prospective studies or RCTs obtain higher scores than cross-sectional or retrospective studies), follow-up data (if any and the length of time), and publication bias (if analyzed and presented). We also added a specific section for the confounding factors considered, which can include controlling for biases, trial quality, and other variables that could influence the results. The magnitude of the impact of the app is determined based on the estimated effect size (the larger the value, the better) [43]. We chose to present effect sizes apart from the quality of evidence for each study. As such, a point is given for each element of the GRADE system measured, with meta-analyses being rated as either very poor, poor, poor to moderate, moderate, moderate to high, high, or very high–quality evidence. No points are given for the effect size. For each meta-analysis, 2 expert raters (TL and SP) rated the different components with the GRADE system. Both raters met and went over their ratings for a final consensus. Given the stringent criteria involved, consensus was easily reached (over 95% initial agreement). For each component of the model, we present both the quality of the evidence and the effect size. Given that meta-analyses also conducted subanalyses, we reported those that compared the apps with a control condition and indicated the results for stand-alone apps versus apps with guidance.

## Results

### Included Studies

Overall, our search retrieved 2558 potential papers. After excluding irrelevant papers and articles that did not respond to our inclusion criteria, we retrieved 24 meta-analyses that were reviewed, of which 7 were included in the meta-review. Please refer to Figure 1 for the flow diagram of the inclusion of meta-analyses in the meta-review.

### Mental Health (Multiple Problems)

Two meta-analyses [44,45] included apps targeting multiple mental health problems, ranging from anxiety, depression, to substance misuse, and even included some studies on physical health problems or stress. The Lindheim et al’s study [44] specifically targeted whether apps offered additional benefits to ongoing treatments or psychotherapy. As such, they only included studies that used apps in addition to a regular (in person) delivered intervention. Overall, the effect size was medium, suggesting that apps can add value to existing treatments. However, the quality of the evidence was rated as poor to moderate (see Tables 1 and 2), given that the effects were imprecise, the samples were very heterogeneous, and the effects were indirect (no subanalyses by diagnosis or problem and all mixed together). However, the meta-analysis included only RCTs, with a total sample size slightly below the criterion of 1000 and verified publication bias. The meta-analysis by Versluis et al [45] was interested in EMI as a tool to increase self-management to cope with depression, anxiety, or stress. As can be seen in Tables 1 and 2, they calculated the effect size in general (all mental health problems together) as well as according to specific outcomes. For the results as a whole, the effect size was medium, the sample size was significant (above 1000), and the publication bias verified, but the other elements did not support quality evidence, with a rating of poor to moderate.

Another meta-analysis also targeted mental health as a larger construct, but within the workplace. This meta-analysis, from the study by Stratton et al [46], however, included various electronic health strategies, with only 3 studies specifically offering an app. The results of these studies are aggregated with other results. As a consequence, the results are indirect, highly heterogeneous, and imprecise with a total quality score of moderate. The effect size was small and decreased when publication bias was included. Nonetheless, the study included a large sample (more than 2000 participants) and only looked at RCTs.

### Anxiety

Versluis et al [45], Firth et al [39], and Linardon et al [47] specifically measured the effect sizes of apps for anxiety symptoms. Although Linardon et al’s [47] meta-analysis is more recent and includes many more studies than the other two meta-analyses, it does not include all the studies found in the two previous studies but has many others, justifying the need to keep all 3 meta-analyses in this review. In a study by Versluis et al [45], a medium effect size was found for EMI on self-management of anxiety symptoms, with poor-quality evidence (because of sample size, heterogeneity of samples, imprecise effect, and no follow-up). The study did, however, look at publication biases and included both RCTs and prospective studies. Firth et al [39], on the other hand, included only RCTs and compared apps with waitlist or active controls and found a small to medium effect size overall when compared with waitlist and small effect when compared with active controls. The meta-analysis included homogeneous samples, samples more than 1000 participants and, overall, were rated of moderate to high quality (but high-quality evidence for the comparison with active controls and waitlists). Finally, Linardon et al [47], focusing on generalized anxiety disorder symptoms, also only included RCTs and considered publication bias (which increased the effect size), and included various controls (waitlist and different types of control conditions: information, placebo/attention, and active controls) and found a small to medium effect size (for all controls together). A closer examination revealed that the effect size decreased as the control condition became more stringent, with the effect no longer being significant when an active treatment control was used. They also looked at some follow-up data and found that the effect size remained small for follow-ups of 2 to 6 weeks. However, those (15 studies) that included follow-ups at 7 to 11 weeks found a medium effect size (g=0.52; 95% CI 0.41 to 0.63). This meta-analysis also considered various subgroup analyses (type of app, intervention model, and specific techniques), but these did not seem to modify the outcome. Overall, we rated this meta-analysis as being of high quality (overall) and moderate quality when compared with active controls because of the strengths mentioned and the fact that the results were either imprecise or inconsistent (or small N for active controls).

#### Specific Anxiety Symptoms

Linardon et al [47] also looked at specific anxiety symptoms, namely, social anxiety, panic, and posttraumatic stress symptoms. Only apps focusing on social anxiety (6 studies) reported a significant medium effect size, with quality evidence of poor to moderate quality (see Tables 1 and 2). Panic and posttraumatic stress symptoms did not improve in the studies reviewed (3 and 4 studies, respectively), with the evidence rated as poor to moderate quality.

### Depression

In total, 3 meta-analyses measured the impact of apps on symptoms of depression and 1 looked at apps for suicidal ideation and self-harm. As was mentioned for anxiety disorders, Linardon et al’s [47] meta-analysis is the most recent but does not include all the studies reviewed in either Versluis et al’s [45] or Firth et al’s [40] meta-analyses, justifying the need to keep all 3 in this meta-review. Versluis et al [45], looking at EMI for self-management of depressive symptoms, found a small to medium effect size, but the quality of the evidence was judged as poor, given the heterogeneity of the samples, the imprecise effect, the study design (no RCTs), the sample size, and the absence of follow-up. The effect was direct, and publication biases were considered. Firth et al [40] compared smartphone interventions with active and inactive controls and only included RCTs. The overall quality of this study was rated as high, with small to medium effect size overall, medium effect size with inactive controls, and small for active controls. Apart from the inconsistency (heterogeneity) and absence of follow-ups, all other quality criteria were met. As for Linardon et al [47], the effect size was small to medium, the effect precise, all studies included were RCTs, a large sample, with no negative effect of publication bias (in fact an increase was noted). Furthermore, follow-ups were reported for some studies, indicating that the effect size was small at posttreatment and at 2 to 6 weeks, but medium at 7 to 11 weeks follow-up (g=0.46, 95% CI 0.36-0.55). The quality of the evidence was also rated as high (overall), given that heterogeneity was found.

Witt et al [48] conducted a meta-analysis on the use of apps for the self-management of suicidal ideation and self-harm. The apps included were solely stand alone. They conducted analyses of suicidal ideation scores, suicidal behaviors, and self-harm behaviors. As can be seen in Tables 1 and 2, when only including RCTs for suicidal ideation, the effect size was small, imprecise, but the sample was homogeneous, followed up with a similar effect size, and the effect was direct. The quality of the evidence was rated as moderate, given the small sample size and the lack of control for biases (publication or otherwise). When looking at noncontrolled studies for suicidal ideation, the quality of the evidence drops to very poor, with small sample size, high heterogeneity, and imprecise effect. As for self-harm, the analyses were mean differences in the frequency of behavior, with nonsignificant effect and poor to moderate–quality evidence.

### Other Mental Health Concepts

Versluis et al [45] also measured the impact of EMI apps on perceived stress, quality of life, acceptance, and relaxation. We chose to only consider perceived stress and quality of life, given that the latter two are theory or intervention specific. Both had small to medium effect sizes with poor quality for perceived stress and poor to moderate–quality evidence for the quality of life (only precision and sample size offered a point). Linardon et al [47] also included indirect measures, namely, distress, stress, and quality of life. They reported a small-medium effect size, but overall, moderate-quality evidence for distress. For stress, the effect size was small to medium but with moderate to high–quality evidence (thanks to various biases controlled for, follow-up data, large sample, and including only RCTs). As for the quality of life, the effect was also small to medium, but the quality of the evidence was high (thanks to precise, consistent effect, large sample, follow-up, and biases controlled for).

Regarding stand-alone apps versus apps offered with guidance or adjunctive to therapy, only some meta-analyses actually compared these, whereas other meta-analyses looked at only one condition. As such, Lindheim et al’s meta-analysis [44] only included adjunctive and had a medium effect size, with poor to moderate quality. Witt et al [48] only included stand-alone apps and found a small effect size, with moderate-quality evidence. Versluis [45] found a medium to large effect size when guidance was offered, compared with medium effect size for stand-alone apps, with stand-alone apps being supported by poor evidence compared with poor to moderate evidence for guidance. Linardon et al’s meta-analysis [47] broke down the guidance versus stand-alone apps according to symptoms targeted (ie, anxiety or depression). For anxiety, the effect size is medium for guidance, compared with small for stand-alone apps, with the quality of the evidence being moderate to high for stand-alone and moderate for apps with guidance. For depression, the effect size was medium for apps with guidance versus small for stand-alone apps, with the quality of the evidence being moderate to high for stand-alone apps and moderate for guidance.

## Discussion

### Principal Findings

This meta-review allowed us to closely examine the quality of the evidence reported by 7 meta-analyses (including various subanalyses) on the use of apps for mental health issues. The results are equivocal, with 14 results being linked to poor or poor to moderate, 15 to moderate or moderate to high, and 8 to high-quality evidence.

When examining studies that include various types of apps for mental health (general), we find that the conclusions are not solid with poor to moderate or moderate–quality evidence, although medium effects (or small effects when looking at work) are reported. For higher quality evidence, samples need to be larger, more homogeneous, with biases and follow-ups included. Although it might be tempting to conduct these larger analyses by merging various apps focusing on different mental health issues, they might not convey quality evidence that is useful.

### Specific Findings

Apps for anxiety symptoms appear to bring a clear benefit of small to medium amplitude, but with good-quality evidence. There are some discrepancies in the results reported, with Firth et al [39] seeing a small effect size when apps were compared with active controls, but in a study by Linardon et al [47], a significant effect was not observed when active controls were used for comparison. This might be because of the inclusion criteria used in these meta-analyses (generalized anxiety symptoms vs anxiety symptoms) or to the much larger sample included in Firth’s analysis. Although follow-ups have only been conducted in a limited number of studies, these report sustained benefits at 6 to 11 weeks. Given that we do not know the frequency of people actually using the apps in the studies (daily, weekly, or less), these results are very promising. The results for specific anxiety problems are of lower quality evidence and did not report a significant clinical effect (for posttraumatic stress disorder or panic disorder), except for social anxiety disorder, which is supported by moderate-quality evidence and a medium effect size. Of import, very few studies focused on apps for specific anxiety disorders.

When looking at apps focusing on depressive symptoms, we obtained small to medium effect sizes compared with waitlist, small when compared with active controls, with overall good-quality evidence (especially for more recent meta-analyses). Furthermore, studies reporting follow-ups show maintenance of the effect at 7 to 11 weeks. These results support the use of apps for depression. The quality of the evidence at this time moderately supports apps for suicidal ideation, with a small effect size but does not support apps for self-harm (no effect).

As for indirect mental health outcomes, namely, outcomes that were considered but were not the main focus of the app intervention (such as distress, stress, or quality of life), the effects are consistently small to medium, with greater quality evidence for the most recent meta-analysis [47].

### Limitations

Our results are limited by its focus on mental health. As such, we did not consider apps that focused on a specific intervention or model (eg, mindfulness apps or CBT) and that did not include symptoms as an outcome. Our results also need to consider what we were not able to measure. Although we sought meta-analyses pertaining to apps in mental health, we did not find meta-analyses for multiple domains or mental health problems for which apps have been developed (eg, severe mental illness, addiction, eating disorders, and obsessive-compulsive disorder). Furthermore, few of the reviews considered confounding factors, such as the actual frequency or time of exposure to the app. Although Weisel et al [41] do not recommend stand-alone apps for mental health problems, our results are more nuanced. Indeed, effect sizes tend to be higher for apps that are used with guidance or with an ongoing treatment (medium effect) compared with stand-alone (small effect), but the quality evidence is better for stand-alone apps. This suggests that stand-alone apps mostly offer a small improvement, but this improvement is consistent across quality studies. As such, apps could be used as a stand-alone treatment, while being on a waitlist for an active treatment, for instance, and offer a small effect on symptoms or offered with some guidance or alongside an ongoing in-person treatment for a medium effect on symptoms.

Furthermore, various studies did not use similar control conditions. The use of different types of control group usually leads to variations in effect estimates. The effect sizes of interventions are typically lower when compared with active controls instead of inactive controls [49,50]. We cannot exclude a digital-placebo effect related to the use of the device itself or from the expectations’ effect [51] rather than from possible active components [52]. Several recent protocols include a placebo intervention (a sham version of the app) [53]; unfortunately, only some of the studies assessed in the included meta-review involved such placebo app control. Furthermore, several studies were conducted with nonclinical populations, who presented with symptoms but perhaps not a diagnosed disorder, limiting the generalizability of the results for clinical populations [54].

Nonetheless, the nature of smartphone interventions does appear to position them as a possible low-intensity intervention tool for those with less severe levels of symptoms or as a first step in a stepped-care approach to service delivery [55]. The follow-up data available to date also suggest that gains are sustainable over a few months. Additional follow-up data are warranted to confirm these results.

Attrition is another problem repeatedly described in smartphone app–related studies [56] and in naturalistic use [57]. Further studies should include a detailed description of the behavior change techniques involved in the design [58] as well as data on the actual utilization of the different app functions. It will be helpful to increase our knowledge about effective strategies in behavior change as well as about the app use engagement. It would also be useful to have a better understanding of the context in which the app is used, at home, at work, at the clinic in the waiting room, alone, or with a therapist or a family member.

### Conclusions

We believe that future studies should focus on high users of apps, namely, youth and young adults. We currently do not have specific information on the efficacy and actual use of mental health apps with such subgroups of individuals. To date, most of the app studies on mental health have focused on feasibility and acceptability, with only a small portion actually pushing forward toward efficacy trials (and often with small numbers). The field of apps for mental health is burgeoning, with the speed of delivery of the app being a primary concern. Traditional study designs (such as RCTs) tend to take a long duration to complete and can deter app developers who aim to commercialize their product. Other controlled research designs could be encouraged (eg, repeated single-case experimental designs) to encourage quality studies at a more rapid speed.

In conclusion, apps for anxiety and depression hold great promise with clear clinical advantages, modestly as stand-alone self-management, and more strongly with guidance or adjunctive treatments. More meta-analyses and more quality studies are needed to recommend apps for other mental health issues or for specific populations.

## Abbreviations

EMI: Ecological Momentary Interventions
GRADE: grading of recommendations, assessment, development, and evaluation
mHealth: mobile health
RCT: randomized controlled trial

## References

[1] Becker S, Miron-Shatz T, Schumacher N, Krocza J, Diamantidis C, Albrecht U (2014). mHealth 2.0: experiences, possibilities, and perspectives. JMIR Mhealth Uhealth.

[2] Bradway M, Carrion C, Vallespin B, Saadatfard O, Puigdomènech E, Espallargues M, Kotzeva A (2017). mHealth assessment: conceptualization of a global framework. JMIR Mhealth Uhealth.

[3] Martínez-Pérez B, de la Torre-Díez I, López-Coronado M (2013). Mobile health applications for the most prevalent conditions by the World Health Organization: review and analysis. J Med Internet Res.

[4] van Singer M, Chatton A, Khazaal Y (2015). Quality of smartphone apps related to panic disorder. Front Psychiatry.

[5] Penzenstadler L, Chatton A, van Singer M, Khazaal Y (2016). Quality of smartphone apps related to alcohol use disorder. Eur Addict Res.

[6] Nicholas J, Larsen ME, Proudfoot J, Christensen H (2015). Mobile apps for bipolar disorder: a systematic review of features and content quality. J Med Internet Res.

[7] Choi J, Noh G, Park D (2014). Smoking cessation apps for smartphones: content analysis with the self-determination theory. J Med Internet Res.

[8] Azar KM, Lesser LI, Laing BY, Stephens J, Aurora MS, Burke LE, Palaniappan LP (2013). Mobile applications for weight management: theory-based content analysis. Am J Prev Med.

[9] Bell IH, Lim MH, Rossell SL, Thomas N (2017). Ecological momentary assessment and intervention in the treatment of psychotic disorders: a systematic review. Psychiatr Serv.

[10] (2017). World Health Organization.

[11] Rahmani AM, Gia TN, Negash B, Anzanpour A, Azimi I, Jiang M, Liljeberg P (2018). Exploiting smart e-Health gateways at the edge of healthcare Internet-of-Things: A fog computing approach. Future Gener Comput Syst.

[12] (2016). International Telecommunications Union.

[13] Owen JE, Jaworski BK, Kuhn E, Makin-Byrd KN, Ramsey KM, Hoffman JE (2015). mHealth in the wild: using novel data to examine the reach, use, and impact of PTSD coach. JMIR Ment Health.

[14] Monney G, Penzenstadler L, Dupraz O, Etter J, Khazaal Y (2015). mHealth app for cannabis users: satisfaction and perceived usefulness. Front Psychiatry.

[15] Kiluk BD, Carroll KM (2013). New developments in behavioral treatments for substance use disorders. Curr Psychiatry Rep.

[16] Sort A, Khazaal Y (2017). Six tips on how to bring epic wins to health care. Front Psychiatry.

[17] Torous J, Hsin H (2018). Empowering the digital therapeutic relationship: virtual clinics for digital health interventions. NPJ Digit Med.

[18] Fatseas M, Serre F, Swendsen J, Auriacombe M (2018). Effects of anxiety and mood disorders on craving and substance use among patients with substance use disorder: an ecological momentary assessment study. Drug Alcohol Depend.

[19] Jones A, Tiplady B, Houben K, Nederkoorn C, Field M (2018). Do daily fluctuations in inhibitory control predict alcohol consumption? An ecological momentary assessment study. Psychopharmacology (Berl).

[20] Merikangas KR, Swendsen J, Hickie IB, Cui L, Shou H, Merikangas AK, Zhang J, Lamers F, Crainiceanu C, Volkow ND, Zipunnikov V (2019). Real-time mobile monitoring of the dynamic associations among motor activity, energy, mood, and sleep in adults with bipolar disorder. JAMA Psychiatry.

[21] Shiffman S, Stone AA, Hufford MR (2008). Ecological momentary assessment. Annu Rev Clin Psychol.

[22] Moore RC, Depp CA, Wetherell JL, Lenze EJ (2016). Ecological momentary assessment versus standard assessment instruments for measuring mindfulness, depressed mood, and anxiety among older adults. J Psychiatr Res.

[23] Torous J, Wisniewski H, Bird B, Carpenter E, David G, Elejalde E, Fulford D, Guimond S, Hays R, Henson P, Hoffman L, Lim C, Menon M, Noel V, Pearson J, Peterson R, Susheela A, Troy H, Vaidyam A, Weizenbaum E, Naslund JA, Keshavan M (2019). Creating a digital health smartphone app and digital phenotyping platform for mental health and diverse healthcare needs: an interdisciplinary and collaborative approach. J Technol Behav Sci.

[24] Neary M, Schueller SM (2018). State of the field of mental health apps. Cogn Behav Pract.

[25] Mantani A, Kato T, Furukawa TA, Horikoshi M, Imai H, Hiroe T, Chino B, Funayama T, Yonemoto N, Zhou Q, Kawanishi N (2017). Smartphone cognitive behavioral therapy as an adjunct to pharmacotherapy for refractory depression: randomized controlled trial. J Med Internet Res.

[26] Berry N, Lobban F, Emsley R, Bucci S (2016). Acceptability of interventions delivered online and through mobile phones for people who experience severe mental health problems: a systematic review. J Med Internet Res.

[27] Firth J, Cotter J, Torous J, Bucci S, Firth JA, Yung AR (2016). Mobile phone ownership and endorsement of 'mHealth' among people with psychosis: a meta-analysis of cross-sectional studies. Schizophr Bull.

[28] Naslund JA, Marsch LA, McHugo GJ, Bartels SJ (2015). Emerging mHealth and eHealth interventions for serious mental illness: a review of the literature. J Ment Health.

[29] Kuhn E, Kanuri N, Hoffman JE, Garvert DW, Ruzek JI, Taylor CB (2017). A randomized controlled trial of a smartphone app for posttraumatic stress disorder symptoms. J Consult Clin Psychol.

[30] Batra S, Baker RA, Wang T, Forma F, DiBiasi F, Peters-Strickland T (2017). Digital health technology for use in patients with serious mental illness: a systematic review of the literature. Med Devices (Auckl).

[31] Velligan D, Mintz J, Maples N, Xueying L, Gajewski S, Carr H, Sierra C (2013). A randomized trial comparing in person and electronic interventions for improving adherence to oral medications in schizophrenia. Schizophr Bull.

[32] Ben-Zeev D, Kaiser SM, Brenner CJ, Begale M, Duffecy J, Mohr DC (2013). Development and usability testing of FOCUS: a smartphone system for self-management of schizophrenia. Psychiatr Rehabil J.

[33] Khazaal Y, Monney G, Richter F, Achab S (2017). Game-control, rational of an application to support the limits of games. Article in French. Jeu-contrôle, rationnel d’une application de soutien aux limites de jeux. J Ther Comport Cogn.

[34] Bertholet N, Godinho A, Cunningham JA (2019). Smartphone application for unhealthy alcohol use: pilot randomized controlled trial in the general population. Drug Alcohol Depend.

[35] Tait RJ, Kirkman JJ, Schaub MP (2018). A participatory health promotion mobile app addressing alcohol use problems (the Daybreak Program): protocol for a randomized controlled trial. JMIR Res Protoc.

[36] Gustafson DH, McTavish FM, Chih M, Atwood AK, Johnson RA, Boyle MG, Levy MS, Driscoll H, Chisholm SM, Dillenburg L, Isham A, Shah D (2014). A smartphone application to support recovery from alcoholism: a randomized clinical trial. JAMA Psychiatry.

[37] Wright CJ, Dietze PM, Agius PA, Kuntsche E, Livingston M, Black OC, Room R, Hellard M, Lim MS (2018). Mobile phone-based ecological momentary intervention to reduce young adults' alcohol use in the event: a three-armed randomized controlled trial. JMIR Mhealth Uhealth.

[38] Crane D, Garnett C, Michie S, West R, Brown J (2018). A smartphone app to reduce excessive alcohol consumption: identifying the effectiveness of intervention components in a factorial randomised control trial. Sci Rep.

[39] Firth J, Torous J, Nicholas J, Carney R, Rosenbaum S, Sarris J (2017). Can smartphone mental health interventions reduce symptoms of anxiety? A meta-analysis of randomized controlled trials. J Affect Disord.

[40] Firth J, Torous J, Nicholas J, Carney R, Pratap A, Rosenbaum S, Sarris J (2017). The efficacy of smartphone-based mental health interventions for depressive symptoms: a meta-analysis of randomized controlled trials. World Psychiatry.

[41] Weisel KK, Fuhrmann LM, Berking M, Baumeister H, Cuijpers P, Ebert DD (2019). Standalone smartphone apps for mental health-a systematic review and meta-analysis. NPJ Digit Med.

[42] Atkins D, Best D, Briss PA, Eccles M, Falck-Ytter Y, Flottorp S, Guyatt GH, Harbour RT, Haugh MC, Henry D, Hill S, Jaeschke R, Leng G, Liberati A, Magrini N, Mason J, Middleton P, Mrukowicz J, O'Connell D, Oxman AD, Phillips B, Schünemann HJ, Edejer TT, Varonen H, Vist GE, Williams JW, Zaza S, GRADE Working Group (2004). Grading quality of evidence and strength of recommendations. Br Med J.

[43] Balshem H, Helfand M, Schünemann HJ, Oxman AD, Kunz R, Brozek J, Vist GE, Falck-Ytter Y, Meerpohl J, Norris S, Guyatt GH (2011). GRADE guidelines: 3. Rating the quality of evidence. J Clin Epidemiol.

[44] Lindhiem O, Bennett CB, Rosen D, Silk J (2015). Mobile technology boosts the effectiveness of psychotherapy and behavioral interventions: a meta-analysis. Behav Modif.

[45] Versluis A, Verkuil B, Spinhoven P, van der Ploeg MM, Brosschot JF (2016). Changing mental health and positive psychological well-being using ecological momentary interventions: a systematic review and meta-analysis. J Med Internet Res.

[46] Stratton E, Lampit A, Choi I, Calvo RA, Harvey SB, Glozier N (2017). Effectiveness of eHealth interventions for reducing mental health conditions in employees: a systematic review and meta-analysis. PLoS One.

[47] Linardon J, Cuijpers P, Carlbring P, Messer M, Fuller-Tyszkiewicz M (2019). The efficacy of app-supported smartphone interventions for mental health problems: a meta-analysis of randomized controlled trials. World Psychiatry.

[48] Witt K, Spittal MJ, Carter G, Pirkis J, Hetrick S, Currier D, Robinson J, Milner A (2017). Effectiveness of online and mobile telephone applications ('apps') for the self-management of suicidal ideation and self-harm: a systematic review and meta-analysis. BMC Psychiatry.

[49] Opriş D, Pintea S, García-Palacios A, Botella C, Szamosközi S, David D (2012). Virtual reality exposure therapy in anxiety disorders: a quantitative meta-analysis. Depress Anxiety.

[50] Pinquart M, Duberstein PR, Lyness JM (2007). Effects of psychotherapy and other behavioral interventions on clinically depressed older adults: a meta-analysis. Aging Ment Health.

[51] Gruszka P, Burger C, Jensen MP (2019). Optimizing expectations via mobile apps: a new approach for examining and enhancing placebo effects. Front Psychiatry.

[52] Torous J, Firth J (2016). The digital placebo effect: mobile mental health meets clinical psychiatry. Lancet Psychiatry.

[53] Giosan C, Cobeanu O, Mogoaşe C, Szentagotai A, Mureşan V, Boian R (2017). Reducing depressive symptomatology with a smartphone app: study protocol for a randomized, placebo-controlled trial. Trials.

[54] Andrews G, Cuijpers P, Craske MG, McEvoy P, Titov N (2010). Computer therapy for the anxiety and depressive disorders is effective, acceptable and practical health care: a meta-analysis. PLoS One.

[55] Marzano L, Bardill A, Fields B, Herd K, Veale D, Grey N, Moran P (2015). The application of mHealth to mental health: opportunities and challenges. Lancet Psychiatry.

[56] Linardon J, Fuller-Tyszkiewicz M (2020). Attrition and adherence in smartphone-delivered interventions for mental health problems: a systematic and meta-analytic review. J Consult Clin Psychol.

[57] Fleming TM, de Beurs D, Khazaal Y, Gaggioli A, Riva G, Botella C, Baños RM, Aschieri F, Bavin LM, Kleiboer A, Merry S, Lau HM, Riper H (2016). Maximizing the impact of e-therapy and serious gaming: time for a paradigm shift. Front Psychiatry.

[58] Edwards EA, Lumsden J, Rivas C, Steed L, Edwards LA, Thiyagarajan A, Sohanpal R, Caton H, Griffiths CJ, Munafò MR, Taylor S, Walton RT (2016). Gamification for health promotion: systematic review of behaviour change techniques in smartphone apps. BMJ Open.

